# Using Impedance Spectroscopy to Assess the Viability of the Rapid Chloride Test for Determining Concrete Conductivity

**DOI:** 10.6028/jres.105.040

**Published:** 2000-08-01

**Authors:** K. A. Snyder, C. Ferraris, N. S. Martys, E. J. Garboczi

**Affiliations:** National Institute of Standards and Technology, Gaithersburg, MD 20899-8120

**Keywords:** building technology, concrete, inductivity, diffusivity, impedance spectroscopy, rapid chloride test

## Abstract

The suitability of using the initial current from the rapid chloride test (ASTM C 1202) to determine specimen conductivity is tested using impedance spectroscopy with a frequency spectrum of 10 Hz to 1 MHz. The specimen conductivity has an analytical relationship to specimen diffusivity and so is a useful quantity in service life prediction. Measurements made on specimens of different lengths indicate that the total charge passed during the six hour conduction test carried out according to ASTM C 1202 is not a direct measure of specimen conductivity. Further, ohmic heating during the 6 hour test makes it nearly impossible to directly measure any specimen transport property from the results. The total charge passed during the 6 hour conduction test is, therefore, not a reliable quantity for service life prediction. Results indicate that the direct current (dc) measurement of resistance using a voltage of 60 V is sufficient to overwhelm polarization effects, thereby yielding an accurate estimate of the true specimen conductivity. Impedance spectroscopy measurements also indicate that corrosion may form on the brass electrodes, adding bias to a conductivity estimate based upon a dc measurement.

## 1. Introduction

There has been considerable research interest in studies of diffusive ion transport through the saturated pore space of portland cement paste. Measurements of conductivity have provided a useful insight into characterizing relevant transport properties [1–8]. This effort has been motivated by the direct relationship between the concrete conductivity and both the steel reinforcement corrosion rate and the ion diffusivity. The concrete conductivity is directly proportional to the reinforcement corrosion current, and it can also be related to the ion diffusivity through the Nernst-Einstein equation for electrical potential gradients or the Nernst-Plank equation for both electrical and chemical potential gradients (see Ref. [9], Chap. 4). Therefore, concrete conductivity can be an important transport property in determining concrete service life in corrosive environments.

Both the American Association of State Highway and Transportation Officials (AASHTO) and the American Society for Testing and Materials (ASTM) have a standardized test of electrical conduction through concrete, referred to here as the rapid chloride test (RCT). This test measures the cumulative electrical charge passing through a specimen subjected to a direct current (dc) voltage of 60 V over a period of 6 h. However, changes in the pore fluid conductivity due to ohmic heating [10,11], and changes in the microstructure due to electromigration [12] prevent the standardized six hour test from yielding a direct measure of specimen dc conductivity and diffusivity.

The work described herein explores whether specimen diffusivity can be determined from some other aspect of the RCT. Experiments have been performed elsewhere that demonstrate the direct relationship between specimen conductivity and diffusivity [13–15]. Experiments have also been performed which purport to demonstrate a causal relationship between measurements of diffusivity and either the total charge passed or the initial current using the RCT cell [16–22]. However, no direct relationships between parameters of the RCT and diffusivity have been proven. Therefore, a means to accurately determine specimen conductivity from RCT data would be a crucial step towards establishing a relationship between RCT data and specimen diffusivity.

Accurate measurements of specimen conductivity can be made using the experimental techniques of impedance spectroscopy (IS). Using an alternating current (ac) that varies over a wide range of frequencies, effects such as electrode polarization and ion transfer can be eliminated, yielding the true specimen resistance. The specimen conductivity may then be calculated from the measured resistance and a knowledge of the specimen geometry. However, under certain conditions, these electrode effects may be negligible. There is a small range of frequencies over which the electrode effects have an insignificant contribution to the overall conductivity, leading a number of investigators to report specimen impedance at a fixed frequency. Further, it may be possible to estimate specimen conductivity in a situation where the electrode effects are relatively small compared to the applied potential. Experiments have shown that the combined electrode effects generate a voltage drop of less than 2 V [3,23]. Therefore, one may conjecture that if a specimen is subjected to a dc electrical potential of 60 V, and if the combined electrode effects generate a negligible voltage drop, the specimen conductivity calculated from the resultant dc current may be sufficiently accurate for most purposes.

Reported herein are the results from IS measurements performed on specimens just prior to the application of the 60 V specified by the RCT. A frequency spectrum of 10 Hz to 1 MHz was used to determine the true specimen resistance, from which the conductivity was calculated. The RCT setup used here recorded the current at 60 s intervals, including the instantaneous initial current, until the completed test at 6 h. Results show that the initial RCT current can be used to directly and accurately determine the specimen conductivity. Implications of this result for possible future “rapid” tests are discussed.

## 2. Impedance Spectroscopy

**Fig. 1:**
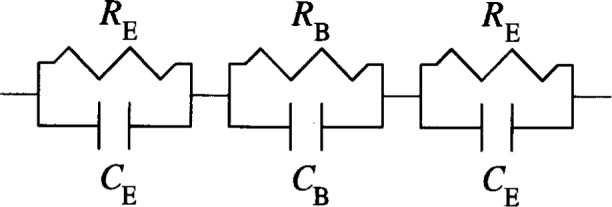
A simplified equivalent circuit used to model the electrode and bulk impedance of the rapid chloride test (RCT) cell. The electrode resistance *R*_E_ and electrode capacitance *C*_E_ characterize the electrode-electrolyte junction. The bulk resistance *R*_B_ and bulk capacitance *C*_B_ characterize the saturated concrete specimen.

Specimen conductivity can be most accurately determined using the principles of IS [24]. In an IS measurement, the specimen is subjected to an ac voltage over a range of frequencies, and the phase (with respect to the applied potential) and amplitude of the current are measured at each frequency. In order to interpret the IS results, the impedance response of a specimen is approximated by an equivalent circuit. The components of the circuit have a physical correspondence to components of the specimen. For metal electrodes against a saturated porous material composed of an insulating solid framework and a pore space filled with electrolyte, the impedance response can be approximated by an equivalent circuit composed of resistances *R* and *C* [24,25]. A simple equivalent circuit for the RCT is shown in Fig. 1. The subscripts “E” and “B” represent the electrode and the bulk components, respectively. The term *bulk* represents the porous solid and electrolyte pore solution composite, and is interchangeable in meaning with *specimen* in this context. The resistance *R*_B_ in Fig. 1 represents the dc resistance of the RCT specimen. The capacitance *C*_B_ represents the specimen capacitance due to the electrolyte in the pore space. The electrode elements *R*_E_ and *C*_E_ represent the impedance response of the electrodes due to polarization and charging effects.

The equivalent circuit in Fig. 1 is composed of parallel resistors and capacitors connected in series. The impedance of a resistor *Z*_R_ and a capacitor *Z*_C_ are complex quantities $\left(i=\sqrt{-1}\right)$ that are parametrized by the ac angular frequency ***ω*** [26]:

$$Z_{R}=R\;Z_{C}=\frac{1}{i\omega C}$$ (1)

The complex nature of the impedance corresponds to a phase difference between the current and the voltage through these devices; a resistor, having a pure real impedance, does not contribute to a change in the phase.

The circuit in Fig. 1 can exhibit both a capacitive and resistive response. For some values of ***ω***, the current and the voltage are nearly in phase. In this case, the impedance has no complex component, and the entire system behaves like a purely resistive element. These values of ***ω*** for which the system is purely resistive can be determined from the total impedance of the equivalent circuit in Fig. 1:

$$Z(\omega )=\left(\frac{2R_{E}}{1+{\left(\frac{w}{w_{E}}\right)}^{2}}+\frac{R_{B}}{1+{\left(\frac{w}{w_{B}}\right)}^{2}}\right)-i\left(\frac{2R_{E}(\frac{w}{w_{E}})}{1+{\left(\frac{w}{w_{E}}\right)}^{2}}+\frac{R_{B}(\frac{w}{w_{B}})}{1+{\left(\frac{w}{w_{B}}\right)}^{2}}\right)=Z^{\prime }(\omega )+iZ^{\prime \prime }(\omega )$$ (2)

The quantities *Z'* and *Z"* represent the real and imaginary components of *Z*, respectively. The constants ***ω***_E_ and ***ω***_B_ are equal to (*R*_E_*C*_E_)^−1^ and (*R*_B_*C*_E_)^−1^, respectively, and are of the same dimension as ***ω*** (i.e., have the same unit). In a system like saturated concrete, the quantity ***ω***_E_ may be several orders of magnitude smaller than ***ω***_B_ (***ω***_E_ ⋘ ***ω***_B_). Given this information, there are three ranges of values for *ω* that are of interest:

$$\begin{array}{l} Z(w\to 0)=2R_{E}+R_{B}\\ Z(w\to \infty )=0\\ Z(\omega_{E}\ll \omega \ll w_{B})=R_{B}. \end{array}$$

The third relationship expresses mathematically the fact that for intermediate values of ω, orders of magnitude from either ***ω***_E_ or ***ω***_B_, the entire system becomes purely resistive; the phase difference between the applied voltage and the resultant current is negligible. Most importantly, this value of *Z* is equal to the bulk specimen resistance *R*_B_ that is used to calculate the specimen conductivity.

**Fig. 2:**
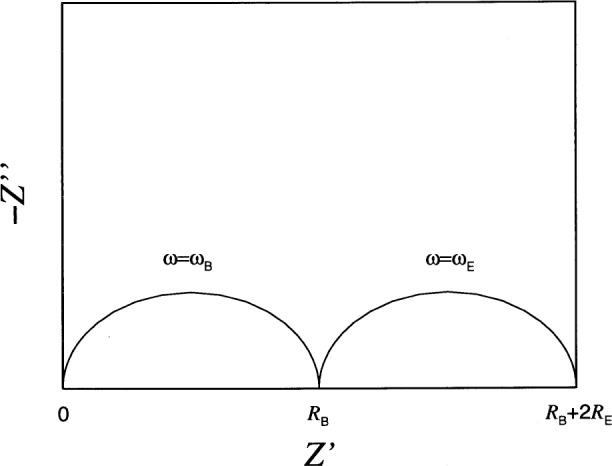
Impedance plane plot showing the real *Z*′ and imaginary *Z*″ components of the total impedance of the circuit in Fig. 1; the independent parameter is the angular frequence *ω*. At the maxima, *ω* is equal to the characteristic frequency of the bulk *ω*_B_ = (*R*_B_*C*_B_)^−1^ and the electrodes *ω*_E_ = (*R*_E_*C*_E_)^−1^, respectively.

**Fig. 3:**
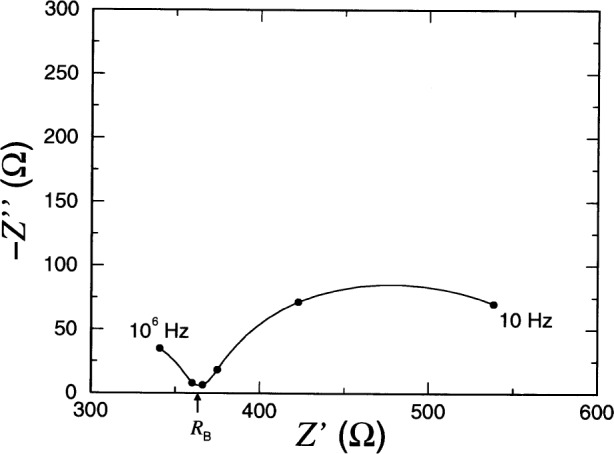
Impedance plane plot showing the real *Z*′ and imaginary *Z*″ components of the total impedance for a typical specimen in the rapid chloride test (RCT) cell; the independent parameter is the angular frequence *ω*. The minimum of the curve is used to determine the sample bulk resistance *R*_B_. Solid circles represent data at decade frequencies; the sampled frequencies ranged from 10^1^ to 10^6^ Hz.

A schematic representation of *Z*(***ω***) is shown in Fig. 2 for ***ω***_E_ ⋘ ***ω***_B_. The figure is an impedance plane plot, typically referred to as a Nyquist plot, and is parametrized by ***ω***, where ***ω*** = 0 is at the right hand side of the curve, and ***ω*** =∞ is at the left. The values of *ω* at the maximum values of −*Z*″ are shown. Experimentally, impedance analyzers can only produce a finite range of frequencies, and typically only the portion of the curve near *Z*′ = *R*_B_ is measured. For an estimate of bulk conductivity, this is all that is required. However, only under ideal conditions does the imaginary component of *Z* go to zero at *Z*′ = *R*_B_. In practice, the response of a specimen is more like that shown in Fig. 3, where the bulk resistance *R*_B_ must be estimated from the value of *Z*′ at the minimum of −*Z*″. The data collected for Fig. 3 consist of 10 data points per decade of frequency. The datum at each decade is shown as a filled circle along the curve. Therefore, the value of *R*_B_ in Fig. 3 was determined at a frequency between 10 kHz and 100 kHz.

**Fig. 4:**
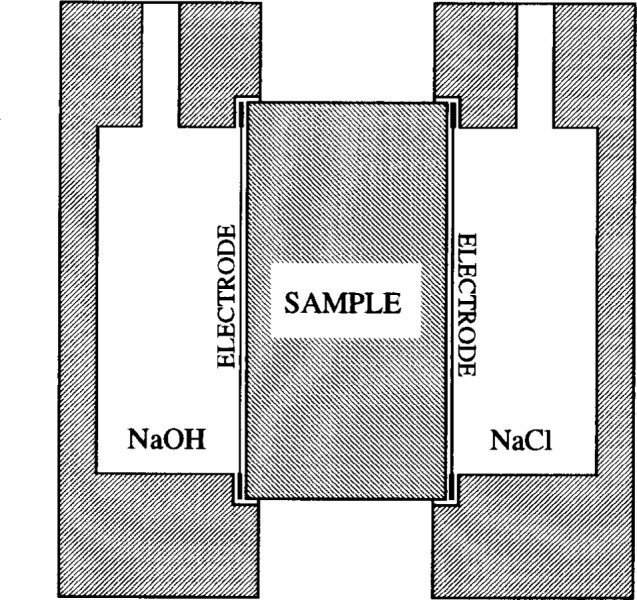
A schematic cross section of the rapid chloride test cell showing the relative positions of the sample and the electrodes. The cylindrical specimen has a diameter of 100 mm and is 50 mm long from electrode to electrode. The solution volume at each end of the cell is approximately 250 mL.

Although a more complete equivalent circuit for the bulk and electrode response of the RCT cell would be more complicated than that shown in Fig. 1 [27,28], this simple circuit captures the major behavior. However, there is an additional component of the bulk impedance that is not represented in Fig. 1. A schematic cross section of the RCT with sample and holders is shown in Fig. 4. Typically, between the specimen and each brass electrode, there is a 1 mm to 5 mm gap that is filled with aqueous electrolyte: either 3 % mass fraction of NaCl or 0.3 mol/L NaOH. The contribution of this resistive component to the total resistance can be calculated from the solution conductivities found in published tables [29]: ***σ***_NaCl_ = 4.4 mS/mm and ***σ***_NaOH_ = 5.7 mS/mm. For the geometry of the RCT cell, the resistances divided by length are 0.029 **Ω**/mm and 0.022 **Ω**/mm for the NaCl and the NaOH solutions, respectively. Therefore, a gap of 10 mm between each electrode and the specimen contributes less than 1 **Ω** to the bulk resistance. Since the bulk resistance for concrete is typically in the range of 100 **Ω** to 1000 **Ω**, the contribution by the electrolyte between the electrodes and the sample can be neglected.

## 3. Experiment

### 3.1 Samples

The mixture proportions of the samples prepared for this experiment were designed to yield a moderate range of transport properties and are based upon mixture proportions from previous experiments [30]. The wide range of transport properties were achieved through variations in water-cement mass ratio *m*_w_/*m*_c_ (commonly denoted by w/c), cement replacement by pozzolanic mineral admixtures, aggregate gradation and volume fraction, and dosage of high range water reducing admixture (HRWRA). The cement was ASTM Type I; the chemical composition is given in Table 1. The pozzolanic mineral admixture was silica fume in slurry form. The aggregate type was natural silica and two gradations were used: silica sand conforming to the ASTM C 778 Graded Sand designation; and a “micro” concrete aggregate composed of natural silica sand. The gradation for the “micro” concrete aggregate, based upon a report by Fuller [31], is shown in Table 2. The HRWRA was an aqueous solution with a naphtalene-sulfonate mass fraction of 40 %. The mixture proportions of the samples used in this experiment are shown in Table 3.

The mixtures were prepared according to the procedures in ASTM C 109. The samples were cast in 100 mm diameter and 200 mm long cylindrical molds, covered, and stored in a 100 % relative humidity chamber. At 24 h of age, the specimens were removed from the molds and stored (50 d to 80 d) in a saturated calcium hydroxide bath until they were tested. Although no temperature controls were used, the laboratory temperature could be characterized by the interval (20 ± 2) °C.

Each specimen was prepared for testing according to the specifications of ASTM C 1202. A single cylinder mold was cast from each mixture and two specimens, one specimen 50 mm long (ASTM C 1202) and the other 100 mm long, taken from the middle 150 mm of the cylinder, were tested. The purpose of using two specimen lengths was to verify the validity of the conductivity measurement techniques. Conductivity, an intrinsic property, is independent of specimen geometry and size. A reliable technique for determining specimen conductivity should obtain equivalent results from replicate specimens with different lengths.

### 3.2 IS and Initial Current Measurements

The specimens were mounted into the RCT cells and the NaOH (0.3 mol/L) and NaCl (3 %) chambers were filled. A commercial impedance analyzer was then connected to the RCT cell banana jacks. Using a voltage of approximately 1 V (peak-to-peak), with a zero volt dc offset bias, across the RCT cell, the analyzer scanned frequencies from 10 Hz to 1 MHz, completing the test in approximately 1 min; results using ac potentials ranging from 0.1 V to 1.0 V yielded similar results. The data were stored in the computer for analysis to determine the value of *R*_B_.

At the completion of the IS measurement, the analyzer leads were removed from the cell and the cell was connected to a commercial dc power supply capable of two-way communication to a computer via an IEEE-488 interface. The computer program would initiate the test, query the power supply for the initial current, and subsequently query the power supply for the current every 60 s until the completion of the test. The power supply was equipped with remote sensing to compensate for the voltage drop along the power cable. This ensured that the voltage delivered to the RCT cell was always within 0.1 V of 60 V.

## 4. Calculated Quantities

The reported values are calculated quantities based upon the physical measurements. For clarity, these calculated quantities shall be defined explicitly. The total charge passed *Q*_T_ is calculated from the measurements of the currents *I_i_* that were carried out at times *t_i_*. Each *t_i_* occurred at the *i*th minute of the test:

$$Q_{T}=\frac{1}{2}\sum_{i=1}^{360}\left(I_{i}+I_{i-1})(t_{i}-t_{i-1}\right).$$ (3)

This method conforms to the procedure specified in ASTM C 1202. The initial RCT resistance *R*_0_ was calculated from the initial current *I*_0_:

$$R_{0}=\frac{60\;V}{I_{0}}.$$ (4)

As indicated in Fig. 3, the bulk resistance *R*_B_ measured using IS was determined from the value of *Z*′ at the minimum value of −*Z*″. Specimen conductivities ***σ***_IS_ and ***σ***_RCT_ were calculated using the measured resistance and the specimen length *L* and area *A*:

$$\sigma_{\mathrm{IS}}=\frac{L}{A\;R_{B}}\;\sigma_{\mathrm{RCT}}=\frac{L}{A\;R_{0}}.$$ (5)

Since a number of studies reported in the literature have used a fixed frequency, it will be instructive to determine the validity of this approach. The quantity *R*_20_ is the real component of *Z*(***ω***) at a frequency of 20 kHz, a frequency that has been used in similar experiments with a fixed frequency [16,18,19].

## 5. Results

The results of the experiment are summarized in Table 4 and are in qualitative agreement with values cited elsewhere [3,17,22,23]. The specimens are grouped by sample for direct comparison between specimens with different lengths from the same mixture. Although most of the specimens were more than two months of age at the time of testing, results of the total charge passed *Q*_T_ indicate that specimens from a range of qualities were tested. In fact, some of the tests had to be terminated due to excessive heating. Unfortunately, there were also some specimens that have no IS data due to computer software problems. However, the comparison among *R*_B_, *R*_20_, and *R*_0_ can be carried out for all but five specimens.

The issue of measurement uncertainty is addressed in Appendix A. Expanded uncertainties *U* reported in Table 4 represent an estimate of two standard deviations, or an estimated coverage of approximately 95 %. The uncertainties in the total charge passed *Q*_T_ were all less than 2 C at this level, with most of the uncertainties less than 1 C. Appendix A addresses the calculations of the reported uncertainties, and concentrates on the complicated issue of the uncertainty in *Q*_T_.

### 5.1 Conductivity

**Fig. 5:**
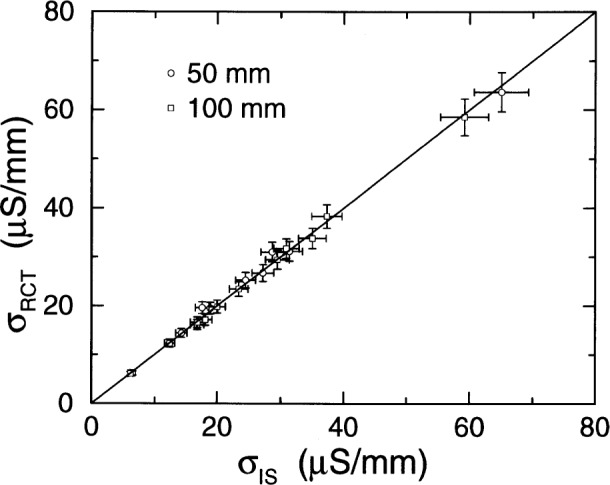
A comparison between the sample conductivity estimated from impedance spectroscopy (IS) and conductivity estimated from the rapid chloride test (RCT) initial current for both the 50 mm and the 100 mm long specimens. The error bars are two standard deviation estimates and the line delineates a 1:1 relationship.

For all but three specimens, the values ***σ***_RCT_ and ***σ***_IS_ for an individual specimen differed by less than 5 %. This fact is demonstrated graphically in Fig. 5. Immediately apparent from graph is range over which the agreement is valid. The data shown on the graph represent samples that passed total charges ranging from 679 C to 10 865 C. In addition, there are data shown that represent specimens that did not finish the 6 h test due to excessive heating.

Since IS gives the most accurate estimate of conductivity, and since the conductivities of replicate specimens of different lengths are not equal, the difference in ***σ***_IS_ between these specimens of different lengths must be due to specimen-specimen variability. Since the values of ***σ***_RCT_ and ***σ***_IS_ for each specimen are nearly equal, the initial current measurements yield a direct and accurate measure of specimen conductivity. Therefore, the ratio of *I*_0_ values for the two specimens is directly proportional to the ratio of specimen conductances (inverse of resistance) However, the ratio of *Q*_T_ values does not equal the ratio of *I*_0_ values. Therefore, *Q*_T_ is not a direct measure of specimen conductivity.

With few exceptions, the resistance measurements at 20 kHz *R*_20_ are within a few percent of *R*_B_. This suggests that there may exist a constant intermediate frequency one could use with the RCT cell to determine sample conductivity to within an acceptable level of uncertainty. However, a suitable frequency should be chosen with care. Using a frequency of 100 Hz, as was used elsewhere [22], would not be advisable due to the substantial contribution by the imaginary portion of the impedance. A fixed frequency test would reduce the cost of an ac test that incorporated the RCT cell. However, with few exceptions, the initial dc resistances are a more accurate estimate than the 20 kHz resistances *R*_20_ measured here.

### 5.2 Total Charge

**Fig. 6:**
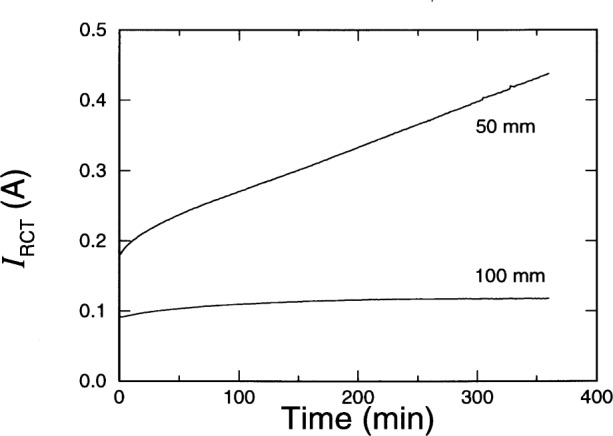
Rapid chloride test current *I*_RCT_ as a function of time for the 50 mm and the 100 mm specimens taken from Sample F.

Although the total charge passed *Q*_T_ increased as the specimen conductance increased, the values are not linearly proportional to one another. Samples for which there are values of *Q*_T_ for both specimen lengths, the values are not inversely proportional to the specimen length; the value of *Q*_T_ for the 50 mm specimen was always significantly greater than twice the value for the 100 mm specimen. Figure 6 shows the measured current for the two specimens from Sample F. The current through the 100 mm specimen is nearly constant throughout the test. The current through the 50 mm specimen appears to increase linearly after approximately 100 min. Although the initial currents differed by a factor of two, the final currents did not. The difference is due to ohmic heating.

Since fluid conductivity increases by approximately 2 % per degree Celsius [10,11], and assuming that specimen pore fluid conductivity behaves in a similar manner, one could estimate sample temperature from the relative change in current. Unfortunately, the specimen temperatures were not measured at the time of the tests and a duplicate experiment that included temperature measurements could not be conducted because the specimens had been discarded.

**Fig. 7:**
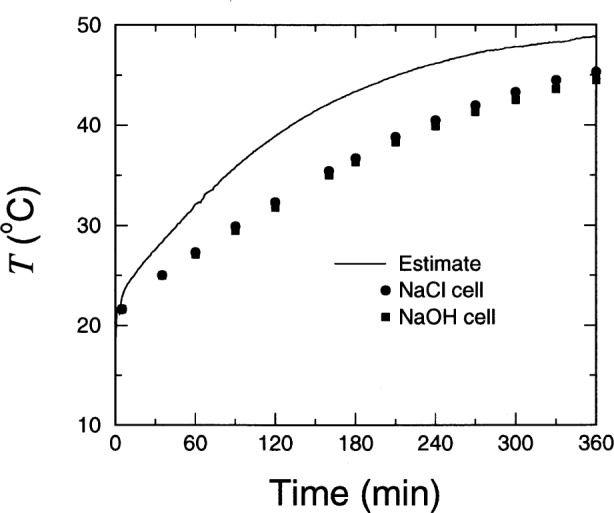
Calculated specimen temperature *T* (solid line) estimated from changes in the rapid chloride test cell current. Measured temperature values (filled circles and squares) were sampled from the solution at each end of the cell.

To demonstrate the effect of temperature, an additional test was performed on a completely different sample from the Federal Highway Administration. For this specimen, the temperature was monitored using an immersion temperature probe incorporating a precision thermistor. The probe was demonstrated to be accurate to within 1 °C. The temperature of the solutions at each end of the specimen was measured periodically throughout the RCT test. The data are shown as filled symbols in Fig. 7. Along with the symbols is a curve that shows the estimated specimen temperature based upon the increase in current passing through the specimen. Although the measured and estimated values are not equal, they agree to within 10 °C throughout the test. This is reasonable since the heat generated within the specimen is lost to the solutions and to the room. Also, the results are in general agreement with laboratory experience and with results reported elsewhere [20,21].

### 5.3 Current at Early Times

**Fig. 8:**
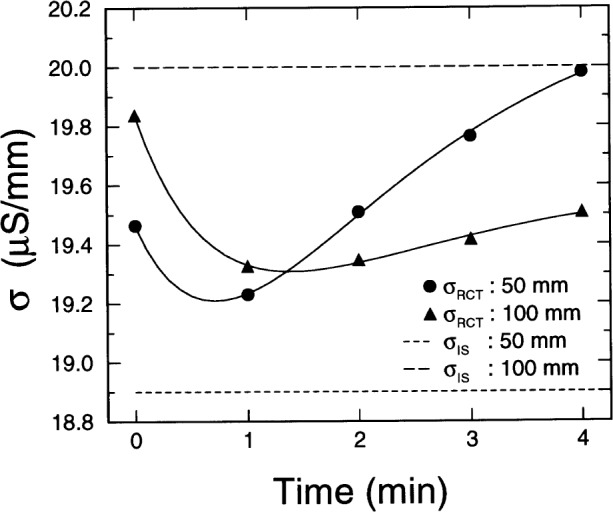
Rapid chloride test (RCT) cell conductivity during the first four minutes of the standard test. The filled symbols were calculated from the measured RCT cell current for both the 50 mm and the 100 mm specimen from Sample F; the solid lines are only meant to guide the eye. The dashed lines are the corresponding RCT cell conductivities as measured using impedance spectroscopy (IS).

It is interesting to note the early conductivity response of the sample to the application of the 60 V required by the standardized RCT. Figure 8 is a plot of effective specimen conductivity calculated from the RCT current for the two specimens from Sample F during the first 4 min of the RCT. The solid lines shown are only meant to guide the reader’s eyes. The dashed lines delineate the sample conductivities using IS. The figure shows the effects of two phenomena: a decrease in current due to the accumulation of polarization charge, and an increase in current due to ohmic heating.

Using these data from Specimen F, it is difficult to distinguish the proper time at which to measure the RCT current to ensure an accurate estimate of *σ*_IS_. One could argue that the proper value should be determined from an extrapolation to zero time using the values measured at 1 min and later. Although this method would be relatively accurate for the 50 mm specimen, it would not be justifiable for the 100 mm specimen. The estimate using the value at *t* = 0 seems to be a reasonable compromise. Since the values in the figure only vary by approximately 4 % during the first 4 min of the test, it would seem as though sufficiently accurate estimates of sample conductivity could be made using the dc current measurements at any time during the first few minutes.

## 6. Discussion

### 6.1 Previous *I*_0_ vs *σ* Data

The direct relationship between the initial RCT current *I*_0_ and sample conductivity established in this experiment is in contrast to results reported elsewhere. Hansen et al. [32] reported both the initial current and the ac impedance at 1 kHz. However, their data showed that, for a given specimen, the ac impedance was considerably greater than the resistance calculated from the initial current. This may be explained using the IS data for the RCT cell that is shown in Fig. 3. The real component of the total impedance at 1 kHz is greater than *R*_B_. Also, if the output of the apparatus used in the experiment by Hansen et al. is the magnitude of the impedance |*Z*| = (*Z*′^2^ + *Z*″^2^)^1/2^, the difference is accentuated by the greater contribution of *Z*″ at 1 kHz than of *Z*′ = *R*_B_. However, the general relationship between initial current and charge passed given in Table 7 of Hansen et al. agree with the results reported here.

The experiments reported by Feldman and coworkers [20,21] also included measurements of specimen impedance and RCT initial current. The impedance measurements were conducted at 10 kHz. However, calculations of specimen resistivity using their RCT initial current differ from the IS measurements by 20 % or more in most cases. The source of the discrepancy cannot be explained by our results since the real component of *Z* at 20 kHz reported in Table 4 is typically within a few percent of *R*_B_.

### 6.2 Significance for Diffusivity

The significance of a rapid test for determining sample conductivity is the relationship between bulk conductivity and bulk diffusivity. The Nernst-Einstein [9] equation can be used to relate the bulk diffusion coefficient *D*_i_ for ion species i to the bulk conductivity *σ*_B_:

$$\frac{D_{i}}{D_{i}^{f}}=\frac{\sigma_{B}}{\sigma_{P}}.$$ (6)

The quantity $D_{i}^{f}$ is the diffusivity of ion species *i* in bulk water, and the quantity *σ*_P_ is the sample pore fluid conductivity. Since the values of $D_{i}^{f}$ can be obtained from tables [10], *D*_i_ could be calculated explicitly from bulk conductivity measurements if the value of *σ*_P_ could be determined using a technique such as the expression of pore fluid [28,33].

For the existing RCT apparatus to be useful to researchers and practitioners, it must be able to measure scientifically useful quantities such as diffusivity or it must be able to report empirical measurements that are directly related to physical processes. It has been shown here that the total electrical charge passed during the RCT 6 h conduction test is not directly proportional to the true dc resistance, and so is not directly related to diffusivity, which is the process of interest. However, the initial current may be used to accurately estimate *σ*_B_.

A second calculation shows that the RCT test does not simulate chloride transport through the sample, and so does not simulate real-world conditions. The magnitude of the drift velocity *v*_D_ of the chloride ions through the RCT cell is calculated from a modification of the Einstein equation [9]:

$$v_{D}=\frac{zeED}{kT}$$ (7)

The relevant quantities are the ion valence *z*, the elementary charge *e*, the magnitude of the applied electric field *E*, the bulk diffusivity *D*, the Boltzmann constant *k*, and the absolute temperature *T*. This equation for the drift velocity *v*_D_ can be used to determine the time required for chloride ions to traverse a specimen, and is in agreement with the experimental results of both McGrath and Hooton [34] and Sugiyama et al. [35].

The drift velocity equation can be simplified using the geometry of the RCT cell (*E* = 1.2 V mm^−1^) and assuming a constant temperature of 300 K:

$$v_{D}=D\times 46.4\;{\mathrm{mm}}^{-1}$$ (8)

This equation can be simplified further by expressing the diffusivity as a ratio of the chloride ion bulk diffusivity at 25 °C (2.0 × 10^−3^ mm^2^ s^−1^ [10]) to the formation factor *F* [36,37]. The chloride ion penetration depth *δ* during the standard 6 h RCT, as a function of the formation factor *F*, is simply the drift velocity *v*_D_ times 21 600 seconds:

$$\delta =\frac{200}{F}\;\mathrm{cm}$$ (9)

Since typical values of the formation factor *F* for 28 d specimens range from 100 to 1000 [38,39], the chloride ions do not traverse the specimen during the rapid chloride test. Therefore, the standard 6 h RCT does not simulate chloride transport through the specimen because the chloride ions typically penetrate only a fraction of the specimen thickness during the test.

### 6.3 Conductivity vs Total Charge Correlations

**Fig. 9:**
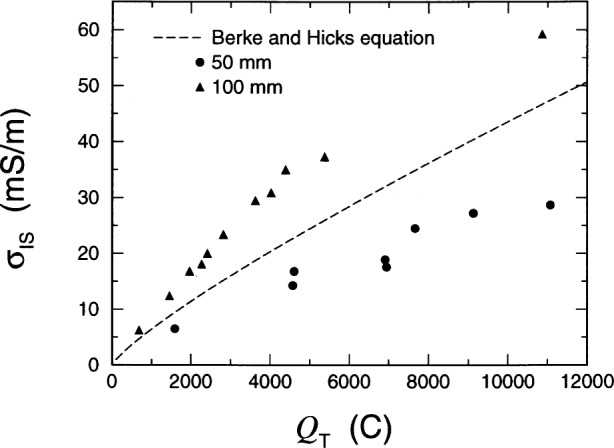
Sample conductivity *σ*_IS_ as measured using impedance spectroscopy (IS) versus the total charge passed *Q*_T_ during the 6 h rapid chloride test (RCT). The filled symbols denote measured data for both 50 mm and 100 mm specimens. The dashed curve is the regression equation of Berke and Hicks [19] for estimating sample conductivity based upon total charge passed during the 6 h test.

Since there have been previous attempts to correlate specimen conductivity to the total charge passed, it will be useful to study this behavior using the data from this experiment. Figure 9 contains a plot of the measured conductivities *σ*_IS_ as a function of the total charge passed *Q*_T_ for the data in this experiment. When considering all the data for a given specimen length, the relationship is not linear over the entire range of *Q*_T_ values. The experiment of Zhao et al. [40] correlated total charge to specimen resistance, but only for specimens passing less than 4500 C. Based upon the data shown in Fig. 9, one would expect a reasonable correlation between total charge passed and specimen conductivity for specimens passing fewer than 4500 C. However, extrapolating a linear correlation for fewer than 4500 C to specimens passing as much as 10 000 C could prove to be erroneous.

**Fig. 10:**
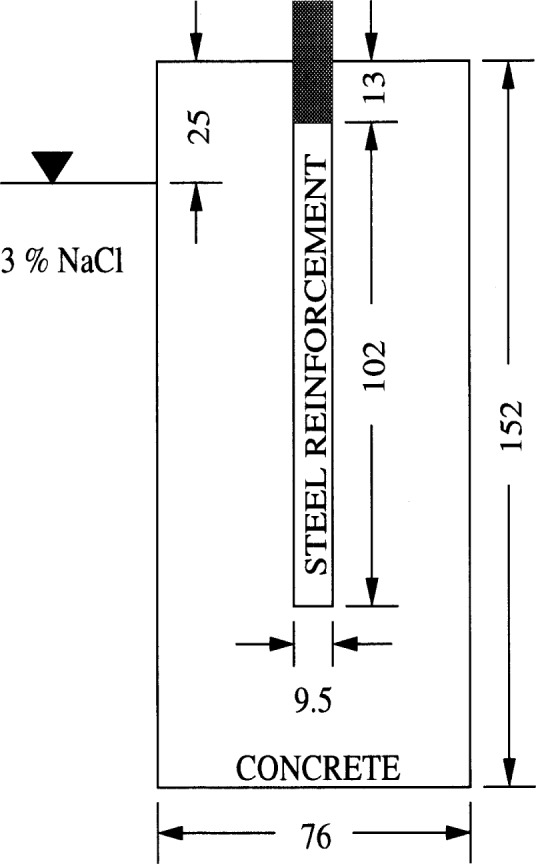
Schematic cross section of *lollipop* apparatus used elsewhere [16,18,19] to measure specimen conductivity. Dimensions shown are in millimeters. The sample is placed into a 3 % mass fraction solution of sodium chloride that contains an additional electrode.

The dashed curve in Fig. 9 represents the empirical prediction of Berke and Hicks (BH) [19] that was developed from correlations between measurements of total charge and conductivity measurements using a *lollipop* apparatus shown schematically in Fig. 10. The apparatus consists of a 9.5 mm diameter reinforcing bar embedded along the axis of a 76 mm diameter, 152 mm long concrete cylinder, with the reinforcing bar positioned 38 mm from the far end of the cylinder. The top 25 mm of the rod penetrating the cylinder is coated with epoxy. Since the BH equation was developed using 50 mm specimens, it should not be expected to predict the response of 100 mm specimens. Also, the equation was developed using data with few values of *Q*_T_ greater than 4000 C. For our experiment, the BH equation is a reasonably good predictor of the 50 mm data for *Q*_T_ less than 4000 C, but is a poor predictor for values of *Q*_T_ greater than 4000 C. This is to be expected, given the parameter space over which the equation was developed. However, there are two features worthy of note. Use of the equation for samples passing greater than 4000 C would introduce large errors. Also, the estimate is consistently greater than the conductivity values measured here. This artifact may be due to the longitudinal component of the current originating from the end of the reinforcement bar used in the BH experiment. This additional current would cause an overestimate of the specimen conductivity, as is demonstrated in Fig. 9.

### 6.4 Corrosion Arc

**Fig. 11:**
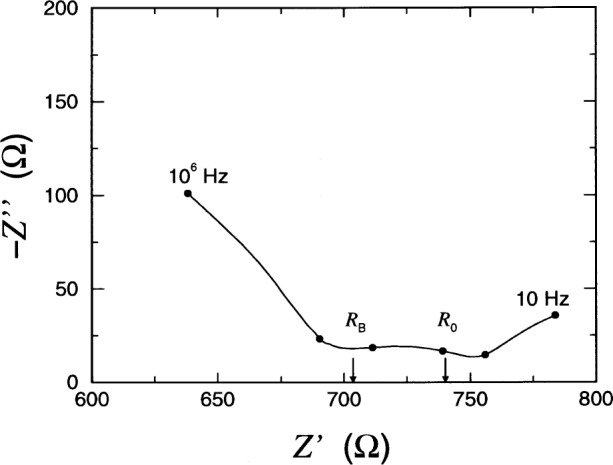
Impedance plane plot showing the real *Z*′ and imaginary *Z″* components of the total impedance for Specimen K-2 in the rapid chloride test (RCT) cell. The independent parameter is the angular frequence *ω*; solid circles represent data at decade frequencies. The specimen bulk resistance *R*_B_ and the sample resistance *R*_0_, which based upon the initial direct current measurement, are shown for comparison purposes.

An interesting component in the Nyquist plots appeared near the completion of this experiment. Samples J through M were cast and tested approximately three months after samples A through I. The IS results from samples A through I were fairly consistent. However, samples J through M exhibited an additional feature in the Nyquist plot such as that shown in Fig. 11 from specimen K-2. The data shown in the figure were measured at the beginning of the RCT, just prior to the application of 60 V dc. The bulk arc can be seen at *Z*′ < 700 Ω, and the electrode arc can be seen at *Z*′ > 750 Ω. The interval 700 Ω < *Z*′ < 750 Ω has an additional arc, possibly due to corrosion accumulating over the duration of the entire experiment. However, the cell was damaged during an attempt to clean one of the brass electrodes in order to confirm the corrosion theory.

The “corrosion” arc presents a possible difficulty in using the RCT cell for determining the sample conductivity. The Nyquist plot of Specimen K-2 in Fig. 11 has two arrows delineating the values *R*_B_ and *R*_0_. The value of *R*_B_ is an accurate estimate of the sample resistance since one would expect the actual value to be at the intercept of the bulk arc and the *Z*′ axis; one can show this by adding another RC pair in the equivalent circuit shown in Fig. 1 to approximate corrosion and observing the result. The measured value of *R*_0_ is affected by the presence of the intermediate arc. Despite this, the values for *σ*_IS_ and *σ*_RCT_ differ by less than 11 % for specimens J through M.

## 7. Summary

The results demonstrated that the total charge passed during the 6 h ASTM C 1202 rapid chloride test (RCT) was not an accurate indication of specimen conductivity. For every mixture proportion studied, the shorter specimen had a disproportionately greater total charge passed. This would be expected based upon the effects of ohmic heating.

A measurement of the initial current (*t* = 0) during the ASTM C 1202 rapid chloride test provides an estimate of specimen conductivity which is typically within 5 % of the value determined from impedance spectroscopy using a frequency spectrum of 10 Hz to 1 MHz. These results were confirmed using otherwise similar specimens of different lengths. Based upon the Nernst-Einstein relationship between specimen conductivity and specimen diffusivity, these results imply that an instantaneous measurement of current can yield quantitative information about the diffusivity of the specimen. Further, data obtained during the start of the RCT suggest that the dc current measured at any time during the first few minutes of the test would yield similar results.

Impedance spectroscopy impedance-plane plots also revealed an electrochemical feature in tests performed later in the experiment. This feature may be attributable to corrosion of the brass electrodes. The feature was responsible for a small bias (less than a 10 % difference) between bulk conductivities measured by impedance spectroscopy and the initial RCT current. Therefore, this suggests that implementing a rapid test based only on the initial current may require frequent monitoring of the electrode surface condition, or the use of electrodes made from a material that does not corrode in the testing solutions.

## Figures and Tables

**Fig. 12:**
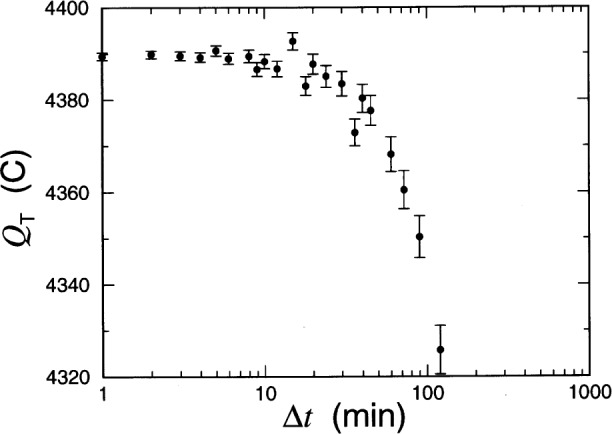
Calculated total charge passed *Q*_T_ through specimen C-2 as a function of the time increment Δ*t* between current measurements. The error bars indicate the *measurement* uncertainty and the variation in the expected values (solid circles) indicate the *method* uncertainty.

**Table 1:** Chemical composition of the Type I cement used in this experiment. The mass fractions not shown are attributable to trace elements

Oxide	Mass fraction (%)
Calcium	63.73
Silica	21.63
Aluminum	4.27
Iron	3.02
Sulfur	2.69
Magnesium	2.26
Free Lime	0.75
Potassium	0.51
Sodium	0.21

**Table 2:** Specification for “Micro” concrete aggregate proportions. Designation C 778 indicates conformity to specification ASTM C 778. The components S15 and F95 are products of the U.S. Silica Company^1^ (Ottawa, IL)

Designation	Range of particle size (µm)	Mass fraction (%)
S15	600 to 2360	37
C 778 (20–30)	600 to 850	19
C 778 (Graded Sand)	300 to 600	19
F95	200 to 300	25

**Table 3:** Sample mixture proportions. Relevant quantities are the sand gradation, the sand to cement mass ratio *m*_s_/*m*_c_, the water to cementitious materials mass ratio *m*_w_/(*m*_s_ + *m*_c_), the fraction of *m*_c_ replaced by silicon, high range water reducing admixture (HRWRA) dosage with respect to cement mass, and the air volume percentage as measured using a procedure based upon the specification ASTM C 185.

Sample	Sand gradation	*m*_s_/*m*_c_	*m*_w_/(*m*_s_ + *m*_c_)	Silica (%)	HRWR (%)	Air (%)
A			0.30			53.7
B			0.45			11.3
C	C 778	2.75	0.60			4.2
D	C 778	2.02	0.36		0.09	6.7
E	Micro	1.41	0.29		0.40	1.2
F	Micro	2.02	0.36		0.09	2.0
G	C 778	2.75	0.49	10	0.40	8.5
H	Micro	2.02	0.36	10	0.40	2.1
I			0.25			2.6
J	C 778	2.75	0.49			8.6
K	C 778	1.40	0.29		0.40	4.2
L	Micro	2.75	0.49			3.9
M	Micro	2.02	0.49		0.09	0.1

Note: Designation C 778 refers to a product conforming to the specification for Graded Sand in specification ASTM C 778.

**Table 4:** Comparison of results between 60 V rapid chloride test (RCT) measurements and impedance spectroscopy (IS) measurements.^a^

Specimen	Length (mm)	Age (d)	*Q*_T_ (C)	*I*_0_ (A)	*R*_0_ (Ω)	*R*_B_ (Ω)	*R*_20_ (Ω)	*σ*_RCT_ (*µ*S/mm)	*σ*_IS_ (*µ*S/mm)
A-1	50	66	b	0.5995	100.1±0.1	98	98	63.6±4.0	65.0±4.3
A-2	100	66	10865±2	0.2755	217.8±0.5	215	213	58.5±3.7	59.2±3.8
B-1	50	73	b	0.9435	63.6±0.1	c		100.1±6.3	
B-2	100	71	b	0.6238	96.2±0.1	c		132.4±8.4	
C-1	50	64	b	0.3622	165.7±0.3	c		38.4±2.4	
C-2	100	64	4389±1	0.1592	377.0±1.5	364	364	33.8±2.1	35.0±2.2
D-1	50	55	b	0.2935	204.5±0.5	203	202	31.1–2.0	31.4±2.0
D-2	100	56	2818±1	0.1103	544.1±3.0	543	540	23.4±1.5	23.4±1.5
E-1	50	69	4564±1	0.1358	441.8±2.0	444	440	14.4±0.9	14.3±0.9
E-2	100	69	1455±1	0.0580	1035±11	1030	1014	12.3±0.8	12.4±0.8
F-1	50	48	6905±1	0.1835	327.1±1.1	336		19.5±1.2	18.9±1.2
F-2	100	49	2418±1	0.0935	641.8±4.2	638	631	19.8±1.3	20.0±1.3
G-1	50	76	4603±1	0.1520	394.7±1.6	379	373	16.1±1.0	16.8±1.1
G-2	100	76	1964±1	0.0781	768.7±5.9	759	738	16.6±1.1	16.8±1.1
H-1	50	75	1591±1	0.0588	1020±11	974	963	6.2±0.4	6.5±0.4
H-2	100	75	679±1	0.0289	2074±43	2050	1992	6.1±0.4	6.2±0.4
I-1	50	69	b	0.1980	303.1±1.0	c		21.0±1.3	
I-2	100	69	3363±1	0.1021	587.9±3.5	c		21.7±1.4	
J-1	50	77	9128±2	0.2514	238.6±0.6	234	228	26.7±1.7	27.2±1.7
J-2	100	77	3625±1	0.1384	433.5±1.9	432	415	29.4±1.9	29.5±1.9
K-1	50	75	6944±1	0.1848	324.6±1.1	362	307	19.6±1.2	17.6±1.1
K-2	100	75	2271±1	0.0807	743.6±5.6	704	704	17.1±1.1	18.1±1.1
L-1	50	78	7662±1	0.2376	252.5±0.7	260	235	25.2±1.6	24.5±1.6
L-2	100	78	4021±1	0.1492	402.1±1.7	412	376	31.7±2.0	30.9±2.0
M-1	50	76	11067±2	0.2921	205.4±0.5	222	193	31.0±2.0	28.7±1.8
M-2	100	76	5373±1	0.1804	332.6±1.1	341	309	38.3±2.4	37.3±2.4

a The RCT resistance *R*_0_ was calculated from the initial RCT current *I*_0_ and the bulk resistance *R*_B_ was determined from impedance spectroscopy. The RCT conductivity RCT was calculated from the RCT resistance *R*_0_ and the IS conductivity IS was calculated from the IS bulk resistance *R*_IS_. The total charge *Q*_T_ passed during the 6 h RCT is the value reported from the standardized test method. The real component of the impedance at 20 kHz *R*_20_ is also shown for comparison purposes. Assigned uncertainties are expanded uncertainties (coverage factor *k* = 2 and hence two deviation estimates); details of the uncertainty evaluation are given in Appendix A.

b Test terminated due to excessive heating.

c No measurement.

**Table 5:** Expanded uncertainties *U* (coverage factor *k* = 2) for the measured quantities voltage *V*, current *I*, current measurement time *t*, specimen length *L*, specimen diameter *D*, and bulk resistance *R*_B_.

Measured quantity	Uncertainty source	*U*
*V*	Equipment specifications	0.054 V
*I*	Equipment specifications	0.0006 A
*t*	Equipment specifications	0.120 s
*L*	Tolerance reported in ASTM C 1202	3 mm
*D*	Tolerance reported in ASTM C 470	0.02 *D*
*R*_B_	Typical nearest neighbor distance at minimum	2.0 Ω

## Footnotes

1 Certain commercial materials are identified in this paper to foster understanding. Such identification does not imply recommendation or endorsement by the National Institute of Standards and Technology, nor does it imply that the materials identified are necessarily the best available for the purpose.

## 8. Appendix A. Evaluation of Uncertainty

### 8.1 Measured Quantities

The evaluation of uncertainty requires assumptions about the magnitudes of the uncertainties attributed to the individual measurements. Unfortunately, none of the individual measurement uncertainties are based upon a statistical analysis. Rather, they are based upon engineering judgment, classified as Type B [41,42] by the International Organization for Standardization (ISO).

Table 5 summarizes the various measurement uncertainties for this experiment. Since the uncertainty in each measured quantity must be quantified, a probability distribution must be chosen for each. Here, a Gaussian distribution is used for convenience. The *uncertainties* are characterized by an expanded uncertainty *U* = *ku*_c_, where *u*_c_ is the standard uncertainty, that is, estimated standard deviation, and *k* is the coverage factor. The expanded uncertainties *U* in Table 5 correspond to two standard deviation estimates (*k* = 2), implying a coverage of approximately 95 %.

The two standard deviation estimates are based upon engineering judgment. If a reputable electronics manufacturer specifies that a voltmeter has an “accuracy” of 0.054 V, it is assumed here that *U* = 0.054 V. This is a conservative estimate because one would generally expect a better than 95 % confidence that the device is within *U* = 2*u*_c_. In Table 5, the values of *U* for the equipment specifications are the accuracies specified by the manufacturer.

Two of the remaining uncertainties in Table 5 are for dimensional measurements. Unfortunately, statistical measurements of the corresponding dimensions of each specimen were not performed. Therefore, the uncertainty in the length and diameter of each specimen is based upon the tolerances specified in the corresponding ASTM specification, with the assumption that the specified tolerance represents a two standard deviation estimate.

The final quantity in Table 5 is the bulk resistance measured by IS. Here, the value of *U* is a “best guess” based solely on general observations. The minimum in the value of –*Z*″ is not an exact quantity. The curvature at the minimum dictates the uncertainty of the determined quantity. The interval defined by *U* represents an overall characterization of the interval between adjacent values of *Z*′ at the minimum of −*Z*″. Many of the adjacent values were considerably less than this quantity, but none was greater.

### 8.2 Calculated Quantities

To obtain the uncertainties of the calculated quantities requires an analysis of both *measurement* uncertainty and *method* uncertainty. The uncertainty in quantities such as the specimen conductivity are based upon standard propagation of uncertainty techniques [42,43]. However, the uncertainty of the total charge passed *Q*_T_ requires an additional analysis of the *method* uncertainty. Equation (3) is not only a means of calculating *Q*_T_, it is also a discrete approximation of the continuous function of current that varies with time. As a numerical method, trapezoidal integration has inherent uncertainty that is a function of both the time interval and the curvature of the function being integrated [44].

Since the curvature in the function of current versus time differs from specimen to specimen, a general approach was needed for the analysis of the method uncertainty. In this experiment, the current through the specimen was measured every minute. From these measurements one can perform a propagation of uncertainty calculation based upon Eq. (3) to yield a measurement uncertainty for a time interval of 1 min. Also, one could extract every other datum, as if the current was measured every 2 min, and perform the same uncertainty calculation. A comparison of these two results would indicate the effect of changing from a time interval of 1 min to a time interval of 2 min. Fortunately, the number 360 (the number of minutes in 6 h) has many possible multiplicative factors. For Sample C-2, the extraction of every *n*th datum was repeated for a number of *n* values, the measurement uncertainty calculated, and the results plotted (see Fig. 12) with the uncertainty bars representing the expanded uncertainty *U*. Based upon these results, for measurement intervals of less than 10 min, there appears to be no significant *method* uncertainty contribution to the overall uncertainty.

## References

[1] Monfore GE (1968). The electrical resistivity of concrete. J PCA.

[2] Whittington HW, McCarter J, Forde MC (1981). The conduction of electricity through concrete. Mag Concr Res.

[3] Hansson ILH, Hansson CM (1983). Electrical resistivity measurements of portland cement based materials. Cem Concr Res.

[4] Atkinson A, Nickerson AK (1984). The diffusion of ions through water-saturated cement. J Mater Sci.

[5] Hope BB, Ip AK, Manning DG (1985). Corrosion and electrical impedance in concrete. Cem Concr Res.

[6] Banthia N, Djeridane S, Pigeon M (1992). Electrical resistivity of carbon and steel micro-fiber reinforced cements. Cem Concr Res.

[7] Feliu S, Andrade C, González JA, Alonso C (1996). A new method for in-situ measurement of electrical resistivity of reinforced concrete. Mater Struct.

[8] Buerchler D, Elsener B, Boehni H, Gerhardt RA, Taylor SR, Garboczi EJ (1996). Electrical resistivity and dielectric properties of hardened cement paste and mortar. Mat Res Soc Proc.

[9] Bockris JO’M, Reddy AKN (1970). Modern Electrochemistry.

[10] Mills R, Lobo VMM (1989). Self-Diffusion in Electrolyte Solutions.

[11] Settle FA (1997). Handbook of Instrumental Techniques for Analytical Chemistry.

[12] Sohn D, Mason TO (1998). Electrically-induced microstructural changes in portland cement paste. Adv Cem Based Mater.

[13] Buenfeld NR, Newman JB (1987). Examination of three methods for studying ion diffusion in cement pastes, mortars, and concrete. Mater Struct.

[14] Andrade C, Sanjuán MA, Recuero A, Rio O (1994). Calculation of chloride diffusivity in concrete from migration experiments, in non-steady-state conditions. Cem Concr Res.

[15] Streicher PE, Alexander MG (1995). A chloride conduction test for concrete. Cem Concr Res.

[16] Scali M, Chin D, Berke NS (1987). Effect of microsilica and fly ash upon the microstructure and permeability of concrete—Proceedings of the Ninth International Conference on Cement Microscopy.

[17] Hansson CM, Berke NS, Roberts LR, Skalny JP (1989). Chlorides in concrete. Mat Res Soc Proc.

[18] Berke NS, Roberts LR, Malhotra VM (1989). Use of concrete admixtures to provide long-term durability from steel corrosion—ACI SP-119. Superplasticizers and Other Chemical Admixtures in Concrete.

[19] Berke NS, Hicks MC, Chaker V (1992). Estimating the life cycle of reinforced concrete decks and marine piles using laboratory diffusion and corrosion data—ASTM STP 1137. Corrosion Forms and Control for Infrastructure.

[20] Feldman RF, Chan GW, Brousseau RJ, Tumida-jski PJ (1994). Investigation of the rapid chloride permeability test. ACI Mater J.

[21] Feldman RF, Prudencia LR, Chan G (1999). Rapid chloride permeability test on blended cement and other concretes: Correlations between charge, initial current and conductivity. Constr Bldg Mater.

[22] Andrade C, Whiting D (1996). A comparison of chloride ion diffusion coefficients derived from concentration gradients and non-steady state accelerated ionic migration. Mater Struct.

[23] Berke NS (1986). Corrosion rates of steel in concrete. ASTM Standardization News.

[24] Macdonald JR (1987). Impedance Spectroscopy.

[25] McCarter WJ, Brousseau R (1990). The ac response of hardened cement paste. Cem Concr Res.

[26] Horowitz P, Hill W (1980). The Art of Electronics.

[27] Scuderi CA, Mason TO, Jennings HM (1991). Impedance spectra of hydrating cement pastes. J Mater Sci.

[28] Christensen BJ, Coverdale RT, Olson RA, Ford SJ, Garboczi EJ, Jennings HM, Mason TO (1994). Impedance spectroscopy of hydrating cement-based materials: Measurement, interpretation, and application. J Am Ceram Soc.

[29] Weast RC (1982). CRC Handbook of Chemistry and Physics, (Indexed by Concen-trative properties of aqueous solutions).

[30] Carino N, Knab L, Clifton J (1992). Applicability of the maturity method to high-performance concrete.

[31] Fuller WB, Thompson S (1907). The laws of proportioning concrete. Trans ASCE.

[32] Hansen MR, Leming ML, Zia P, Ahmad S, Zia P (1993). Chloride permeability and ac impedance of high performance concrete—ACI SP-140. High Performance Concrete in Severe Environments.

[33] Barneyback RS, Diamond S (1981). Expression and analysis of pore fluids from hardened cement pastes and mortars. Cem Concr Res.

[34] McGrath PF, Hooton RD (1996). Influence of voltage on chloride diffusion coefficients from chloride migration tests. Cem Concr Res.

[35] Sugiyama T, Tsuji Y, Bremner Theodore W, Hashi-moto C, Malhotra VM (1996). Determination of chloride diffusion coefficient of high-performance concrete by electrical potential technique—ACI SP-163. Concrete in Marine Environment.

[36] Wong P (1988). The statistical physics of sedimentary rock. Phys Today.

[37] Schwartz LM, Banavar JR (1989). Transport properties of disordered continuum systems. Phys Rev B.

[38] Garboczi EJ, Bentz DP (1992). Computer simulation of the diffusivity of cement-based materials. J Mater Sci.

[39] Shane JD, Mason TO, Jennings HM, Garboczi EJ, Bentz DP Conductivity of the interfacial transition zone in portland cement mortars.

[40] Zhao TJ, Zhou ZH, Zhu JQ, Feng NQ (1998). An alternating test method for concrete permeability. Cem Concr Res.

[41] (1993). Guide to the Expression of Uncertainty in Measurements.

[42] Taylor BN, Kuyatt CE (1994). Guidelines for Evaluating and Expressing the Uncertainty of NIST Measurement Results.

[43] Wilson EB (1952). An Introduction to Scientific Research.

[44] Gerald CF, Wheatley PO (1984).

